# How to assess the effectiveness of nasal influenza vaccines? Role and measurement of sIgA in mucosal secretions

**DOI:** 10.1111/irv.12664

**Published:** 2019-06-21

**Authors:** Elena Gianchecchi, Alessandro Manenti, Otfried Kistner, Claudia Trombetta, Ilaria Manini, Emanuele Montomoli

**Affiliations:** ^1^ VisMederi Srl Siena Italy; ^2^ VisMederi Research Srl Siena Italy; ^3^ Department of Molecular and Developmental Medicine University of Siena Siena Italy

**Keywords:** enzyme‐linked immunosorbent assay, influenza vaccines, influenza virus, mucosal immunity, secretory IgAs

## Abstract

Secretory IgAs (sIgA) constitute the principal isotype of antibodies present in nasal and mucosal secretions. They are secreted by plasma cells adjacent to the mucosal epithelial cells, the site where infection occurs, and are the main humoral mediator of mucosal immunity. Mucosally delivered vaccines, such as live attenuated influenza vaccine (LAIV), are able to mimic natural infection without causing disease or virus transmission and mainly elicit a local immune response. The measurement of sIgA concentrations in nasal swab/wash and saliva samples is therefore a valuable tool for evaluating their role in the effectiveness of such vaccines. Here, we describe two standardized assays (enzyme‐linked immunosorbent assay and microneutralization) available for the quantification of sIgA and discuss the advantages and limitations of their use.

## 1 INTRODUCTION

Influenza is a disease with high morbidity and mortality which is caused by influenza viruses of types A and B.^1^, ^2^ Seasonal influenza epidemics are estimated to result in 3‐5 million cases of severe illness and about 290 000‐650 000 respiratory deaths worldwide.^2^

## 2 SECRETORY IgA PRODUCTION UPON NATURAL INFLUENZA INFECTION

**Figure 1:**
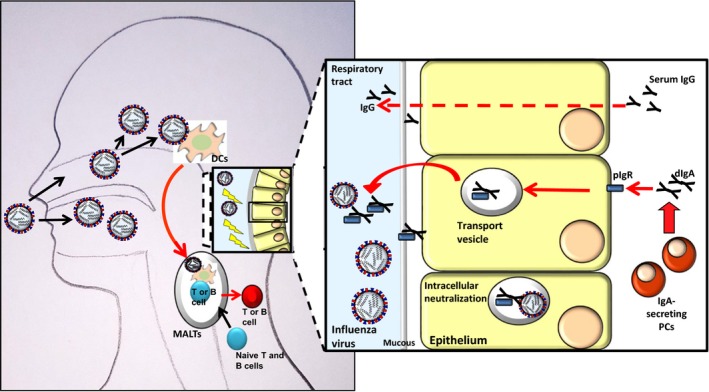
Simplified scheme of immune responses following influenza virus infection of the upper respiratory tract with focus on induction and mode of action of secreted IgA (sIgA). Abbreviations: B cell, B lymphocyte; DCs, dendritic cells; dIgA, dimeric IgA; IgA, immunoglobulin A; IgG, immunoglobulin G; MALT, mucosa‐associated lymphoid tissue; PCs, plasma cells; pIgR, polymeric immunoglobulin receptor (pIgR); T cell, T lymphocyte. Influenza viruses infect epithelial cells of the mucosa and induce mucosal immune responses. Mucosal immune system consists of two sites. Inductive site (MALT) for antigen uptake by DCs and priming of T and B cells for IgA antibody production. Effector site with IgA‐secreting PCs. DCs take up exogenous virus antigens (from virus particles or apoptotic infected cells) by endocytosis and activate naïve T and B cells. PCs secrete IgA antibodies. IgG antibodies transude from the serum to the mucus by diffusion and provide protection against homologous influenza viruses. dIgA are actively transcytosed across epithelial cells via pIgR and provide protection against homologous and heterologous influenza viruses. dIgA can bind to newly synthesized viral proteins within virus‐infected epithelial cells and prevent virus

Influenza viruses infect humans through the mucosal epithelium covering the upper respiratory tract; thus, the respiratory epithelium constitutes the site of virus entry, infection, and host immune response (Figure 1). Antibodies located on the surface of the mucosa represent the major immune components providing protection against influenza. Upon infection, the human humoral immune response is activated leading principally to the production of local secretory IgA (sIgA) in the mucosa of the upper respiratory system, serum IgA and IgG antibodies.^3^

### 2.1 Role of IgA and IgG antibodies

Whereas sIgA antibodies have the role to neutralize potential pathogens at the entrance site before they could attach epithelial cells and overcome the epithelium surface, serum IgA represent “backup” antibodies whose function is to respond in case of systemic infection due to invasion across the mucosal epithelium.^4^ IgG‐secreting cells are produced in the mucosa‐associated lymphoid tissues (MALTs) and regional lymph nodes. IgG antibodies are secreted in the bloodstream and, reached the mucosal tissues, move via diffusion from the serum to the mucus.^5^ IgA is characterized by an elevated ability in preventing virus infection, whereas IgG exerts only a marginal role in providing protection toward infections affecting the upper respiratory system. However, IgG antibodies are principally involved in reducing viral pneumonia.^6^

### 2.2 Role of cellular immune responses

Along with the humoral immunity, also the cell‐mediated immune (CMI) response is activated after influenza infection. Unlike humoral response, capable of neutralizing activity, CMI is able to prevent virus replication and decrease the time for recovery.^7^, ^8^ CD4^+^ follicular helper T (Th) lymphocytes in presence of antigen‐presenting cells (APCs), such as dendritic cells (DCs) and influenza antigens, induce the differentiation of naïve B cells into IgA‐secreting plasma cells (PCs). sIgA constitute the principal isotype of antibodies present in external secretions, such as nasal fluid, saliva, milk, colostrum intestinal fluid, and gallbladder bile.^9^ In the upper respiratory tract, sIgA antibodies are secreted by mucosal PCs adjacent to the mucosal epithelial layer at the site of infection^10^ and represent the main humoral mediator of nasal immunity.^11^

### 2.3 Immune mechanisms contributing to disease reduction or protection

In influenza‐naive subjects, the clearance of primary viral infection occurs through sIgA and cytotoxic T lymphocytes (CTLs). More specifically, sIgA appear on day 5 post‐infection, and their level rapidly increases in the nasal wash until day 7‐10 post‐infection, when it reaches a plateau. IgA local immune response persists for a period of 3‐5 months^5^, ^12^, ^13^ and then gradually diminishes returning to the pre‐immunization levels within 6 months.^14^ In addition, it is possible to detect IgA‐producing memory cells locally.^5^, ^12^, ^13^ CTLs appear transiently in the nasal mucosa and peak on day 7 after infection. sIgA have a pivotal role in protecting against influenza infection of the upper respiratory mucosal surfaces, since they can disarm the virus either before it crosses the mucosal barrier^15^ or in infected epithelial cells by intracellular neutralization.^15^, ^16^ The magnitude of the IgA antibody response is directly correlated with resistance to new infections.^17^ In addition, IgA is the predominant Ig isotype in local secretions after secondary infection and an IgA response is also detected in the serum upon subsequent infection which support its additional important role in protection against influenza virus re‐infections.^18^

Along with IgA, IgM antibodies are also secreted actively across the mucosa and may contribute to protection by preventing viral entry into the cells and also interfering with virus replication in the cells.^5^, ^18^ The potential protective role of IgM antibodies is supported by a study in mice which has shown that IgM antibodies can neutralize influenza viruses in the presence of complement just as well as IgG antibodies.^19^

### 2.4 IgA immune responses upon influenza virus infection of the mucosa of the upper respiratory tract

On the basolateral surface of the epithelial cells in the lamina propria of mucosal tissue, a polymeric Ig receptor (pIgR) links the dimeric IgA (dIgA) and moves to the apical side (Figure 1). During the process of transcytosis across the epithelial cells, polymeric IgA (pIgA) acquires the secretory component (SC), producing sIgA. Secretory component is an unusual extra polypeptide that constitutes the extracellular portion of pIgR upon cleavage by a selective protease.^20^ The presence of SC provides sIgA a greater functional stability, both by masking the protease sites from proteolytic degradation operated by proteases present in mucosal secretions^21^ and by sustaining the association of monomeric IgA (mIgA).^22^

IgA do not promote the activation of the inflammatory complement system, a feature which is critical to maintaining the integrity of the mucosal barrier.^23^

### 2.5 Presentation forms and functions of IgA antibodies

In human serum, IgA are mainly present in the monomeric form with two α‐heavy and two light chains. On the other hand, in external secretions IgA are highly heterogeneous in terms of their quaternary structure but the majority are in polymeric form.^24^ sIgA are generally present as a dimer, despite, and at low frequency, as larger polymeric forms (pIgA) especially tetramers.^25^

It has been hypothesized that pIgA may have a higher ability than mIgA to neutralize intracellular viral particle assembly by binding newly synthesized viral proteins.^5^, ^17^, ^26^ It has also been demonstrated that the polymeric nature of sIgA was responsible for their elevated cross‐reactivity, thereby increasing the avidity of this antibody subclass in comparison with mIgA and serum IgG.^27^, ^28^

The best neutralizing activity and the higher avidity of human pIgA than mIgA can be attributed to the presence of multiple antigen‐binding sites located on each Ig polymer, indicating that the quaternary structure plays a key role for their potency.^25^ This result is in accordance with previous researches conducted on mice.^27^, ^29^, ^30^ A recent study by Saito and colleagues demonstrated that IgA tetramerization improves target breadth exerting no effect on potency of functionality of anti‐influenza virus broadly neutralizing antibody.^31^ The higher anti‐viral activity of pIgA than mIgA is particularly important, considering the anatomical site of sIgA action.^10^ pIgA appears to have a greater inhibitory potential in preventing viral attachment and virus neutralization than mIgA and also IgG.^29^, ^32^, ^33^ Another study showed the existence of larger pIgA in addition to tetrameric sIgA in the upper respiratory tract. The proportion of this polymeric form is approximately 20% of the total IgA.^34^

In summary, the mucosal surface is endowed with two protective barriers against viral infection, both of which involve mucosal IgA, that is, extracellular sIgA and intracellular pIgA.^29^

## 3 IgA IMMUNE RESPONSE UPON INFLUENZA VACCINATION

Conventional inactivated influenza vaccines (IIVs), generally delivered through subcutaneous or intramuscular injection, are today still the most efficient, valuable, and low‐cost tools to effectively reduce influenza virus infections and subsequently morbidity and mortality.^35^ This parental administration is able to increase the serum antibody level in the systemic immune compartment, but it is not able to trigger a local mucosal immune response at the site of primary infection, that is, an induction of sIgA which exhibit a wide cross‐protection activity. This represents a limit for conventional inactivated influenza vaccines in conferring full protection against infection.^36^

While natural infection is able to induce both mucosal and systemic heterosubtypic responses, the immunity induced by parenterally application of inactivated influenza vaccines is generally virus subtype‐specific.^37^ In pre‐immunized subjects, the natural contact with the pathogen causes a rapid synthesis of IgA and IgG by B memory cells already 3 days after infection. These immunoglobulins form Ig‐virus complexes which result in virus inactivation.^17^

In recent years, an increasing number of pre‐clinical and clinical studies have been performed which have led to a better understanding how mucosal antibodies could be elicited by intranasal vaccination with live attenuated influenza vaccines (LAIV). Live attenuated influenza vaccines is administered as a nasal spray and contains a cold‐adapted (ca) live attenuated influenza virus which, in contrast to wild‐type viruses, is able to replicate well at lower temperatures (around 25°C) and as such only in the upper respiratory tract, but not at higher temperatures (37°C) which does not allow replication in the lower respiratory tract including the lungs. Such property does not allow the ca virus to replicate in lung tissues or cause the onset of influenza‐like illness.^38^ LAIVs have been introduced firstly by Russia.^39^ They have been used in adults since the 1950s, and from 1987 onwards, the use of Russian LAIV for the prophylaxis of influenza has been widely extended to all age groups including children aged over 3 years.^40^ LAIVs have been licensed in the Unites States (US) in 2003 for healthy subjects aged 2‐49 years and in the European Union (EU) in 2012 for healthy children aged 2‐17 years.^41^ Whereas some countries, including Russia, have licensed only trivalent LAIVs (T‐LAIVs), recently a quadrivalent LAIV (Q‐LAIV) vaccine (MedImmune/AstraZeneca) has been introduced in other countries, such as the US (since 2012), Canada (since 2013), and EU (since 2015), under the trade names FluMist™ in the US and Canada, and Fluenz^R^ in the EU.^42^

Studies performed in mice have demonstrated the predominant protective role played by sIgA,^43^, ^44^ even in case of absence of T cells.^45^ Specifically, the passive intranasal transfer of anti‐influenza A IgA from the respiratory tract of mice immunized with live influenza virus has been seen to provide protection in naive mice.^43^ Accordingly, this protection was suppressed by the intranasal instillation of anti‐IgA,^46^ whereas it was not affected by treatment with anti‐IgM or anti‐IgG antibodies. This result supports the importance of IgA as a mediator of murine nasal anti‐influenza virus immunity in immunocompetent mice.^47^

Furthermore, several studies have found a higher level of correlation between the degree of protection and the antibody secretory level than serum antibodies both in mice^48^ and in humans.^5^

T‐LAIVs have been widely investigated in several clinical studies conducted on different age cohorts throughout the world.^49^ After influenza LAIV administration, as well as natural infection, sIgA are produced by memory B lymphocytes.

The immunogenicity, efficacy, or effectiveness of LAIVs in comparison with IIVs has been analyzed in a number of studies.^50^, ^51^, ^52^, ^53^, ^54^ A meta‐analysis of a number of investigations has shown that the LAIV has been less effective than IIV in general.^55^ However, individual studies with respect to mismatched vaccine strains or in children with underlying diseases such as asthma have shown a higher efficiency in comparison to IIV, for example, with respect to a circulating variant (A/Sydney/H3N2) not present in the vaccine composition with an efficacy of 86% against this mismatched circulating strain^51^ or in children affected by asthma^53^ or recurrent respiratory tract infections.^52^

A more recent study conducted by McLean^54^ reported a similar effectiveness provided by a Q‐LAIV and IIV against a new antigenic A(H3N2) variant, whereas a considerable higher protection was provided by Q‐LAIV compared to IIV toward a drifted influenza B strain.

Data obtained from the US reported an apparent lack of LAIV effectiveness in the 2015/2016 influenza season, especially toward A/H1N1 vaccine component. Such results have led the Advisory Committee on Immunization Practices (ACIP) not to use the LAIV in the US during the 2016/2017 seasons.^56^ Conversely from the observations reported in the US, a higher overall protection of the LAIV against laboratory‐confirmed infection with the A/H1N1 strain in comparison with IIV has been reported by the UK^57^ and Finland in the 2015/2016 season.^58^ For these reasons, LAIV use is closely monitored, but it is still recommended in these countries as well as in Norway, although the motives underlying this difference have not been elucidated yet.

The persistence of protective mucosal immune responses upon LAIV immunization has been investigated in several studies. Murphy and Clements^45^ found elevated levels of IgA that recognize HA, and reduced levels of IgM and IgG, in nasal washes obtained from naïve children infected 2 weeks earlier by means of attenuated A viruses. In about 50% of the vaccinees, IgA and IgG in the nasal wash persisted for 1 year. Subsequent studies confirmed the persistence of long‐term (at least 1 year) immunological memory following LAIV vaccination^59^ and of serum IgG.^6^ The longevity of local immune response up to a year indicates that the mucosal immune system is well developed also in young children.

Clinical Studies have shown that protection after LAIV vaccination is correlated with local anti‐hemagglutinin (HA) IgA and anti‐neuraminidase antibodies in serum,^6^ whereas IgG antibodies are the main effectors in providing protection in the mucosal compartments of human vaccinated with inactivated vaccines. These antibodies derive from plasma through a process of passive transudation following a concentration gradient between plasma and nasal IgG.^60^ The substantial differences in the presence of anti‐viral IgA and IgG antibodies in nasal washes or serum of individuals vaccinated either with LAIV or with IIV indicate that these two vaccines are inducing fundamentally different immune responses resulting in different mechanisms of protection. Whereas the protection of the upper respiratory tract is provided mainly by IgA with IgG playing a minor part, the latter play a pivotal role in the protection of the lungs.^6^, ^60^, ^61^, ^62^ These differences in the specific immune responses have been confirmed by the recent meta‐analysis of the group of Wen et al^62^ which identified 191 and 195 differentially expressed genes in IIV and LAIV recipients, respectively. Whereas IIV induced the up‐regulation of genes associated with both the innate immune response and the humoral immune response, LAIV mostly elicited the innate immunity.

These data suggest that intranasal vaccinations may be the best choice to achieve immune responses which mimic natural infections by stimulating both systemic and mucosal immune response,^63^, ^64^ but without causing the signs and symptoms associated with influenza illness.^49^ Nowadays, intranasal vaccination against influenza is mainly made up through ca LAIVs.

However, other alternative ways used to induce mucosal immunity are currently available. These include the following: intranasal vaccinations using inactivated whole or split influenza vaccines*,* 65, 66 sublingual administration of adjuvanted influenza vaccines,^67^ and novel types of LAIVs, for example, formulated by depleting the *NS1* gene. *NS1* encodes for a non‐structural protein, resulting in attenuated viruses (DelNS1 viruses) unable to overcome the anti‐viral defenses of infected cells.^37^, ^68^

## 4 ELISA ASSAYS FOR THE DETERMINATION OF THE IgA CONTENT IN TEST SAMPLES

According to the type of influenza vaccine used and the route of administration, specific compartments of the human immune system are stimulated. A local mucosal immune response is elicited by natural infection or intranasal vaccination, while a systemic immune response develops after parenteral vaccination.^69^ As a consequence of stimulating different components of the immune system via mucosal or parenteral application of vaccines, the induction of serum hemagglutinin inhibition (HI) antibody titers, which are still considered as the gold standard in assessing influenza vaccine immunogenicity, is generally lower after intranasal vaccination than those elicited by intramuscular vaccination^50^; conversely, high levels of nasal IgA have been observed in recipients of LAIV.^63^ Of out a couple of laboratory tests that can be used to assess influenza antibody levels, the enzyme‐linked immunosorbent assay (ELISA) is the most favorable one to measure the mucosal immune responses.

Enzyme‐linked immunosorbent assay can accurately measure the concentrations of different classes of antibodies that are able to bind to influenza virions or purified HA proteins.^70^ Many different protocols and standard kits are currently available on the market, but they are all based on the same principle. Generally, HA protein or whole influenza virus is pre‐adsorbed to the wells of an ELISA microplate; different sample dilutions are then added, followed by the addition of the labeled secondary antibody, which is able to detect the immunoglobulins of interest. A colorimetric reaction is obtained upon the addition of a substrate. An important feature of the ELISA assay is that it can measure different classes of IgG, IgM, and IgA present both in serum and in mucosal samples.^71^, ^72^, ^73^

**Figure 2:**
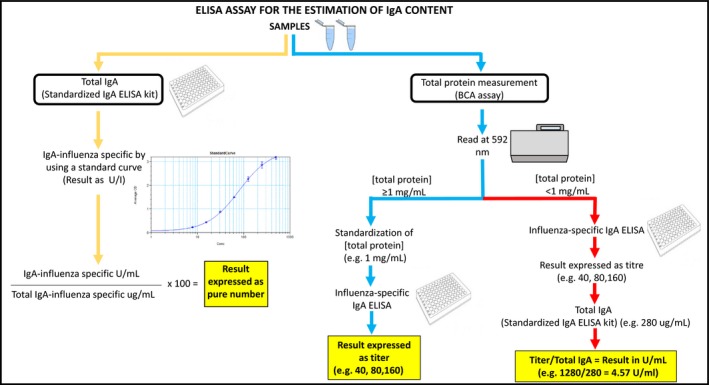
Principles of ELISA assays for the determination and standardization of the IgA content in test samples. Test samples include nasal washes, swabs, or saliva samples. Method 1: Standardization of antigen‐specific IgA antibodies against total IgA (left side, yellow arrows). Method 2: Standardization of antigen‐specific IgA antibodies against total protein (right side, blue and red arrows) Variation 1: high total protein content (>1 mg/mL; middle right side, blue arrows). Variation 2: low protein content (<1 mg/mL) or big differences of the total protein content of different test samples (rightmost side, red arrows)

Currently, there are two main methods (Figure 2) of detecting influenza‐specific sIgA responses in nasal wash/swab and saliva samples.^74^, ^75^ The two methods differ mainly in terms of the strategy adopted for the standardization of the samples to be analyzed, which precedes influenza‐specific IgA detection. Standardization of the mucosal samples is an important step, since the mucus and protein concentration of nasal washes varies widely between individuals, depending on several factors, such as age, history or concurrence of nasal disease, and aspiration efficacy.^14^ The first method described here is based on sample standardization according to the total content of IgA present in each sample by using a standardized IgA ELISA kit, whereas the second method is based on the quantification of total protein content through the bicinchoninic acid (BCA) assay (Figure 2).

### 4.1 Influenza HA‐Specific IgA respect to Total IgA

This method is used to normalize the influenza‐specific IgA content of a sample through the total IgA content (Figure 2). The total IgA concentration in nasal wash/swab samples or saliva can easily be measured by using one of the many standardized ELISA kits available on the market. Concerning influenza‐specific IgA detection, the procedure needs to be adapted due to the absence of a standardized human influenza‐specific IgA reference. The ELISA procedure in principle has been described elsewhere.^73^ In brief, ELISA plates are coated with an influenza antigen (preferably purified HA) and a capture antibody (anti‐human IgA). Samples and standards are added to the plate and incubated for 1‐2 hours at 37°C. The presence and concentration of influenza‐specific IgA or total IgA is then determined by a color reaction applying an enzyme‐labeled second antibody against human IgA and the respective substrate followed by a read‐out in a conventional microtiter plate ELISA reader. The anti‐influenza IgA concentration is being extrapolated from the standard regression curve derived by diluting a human total IgA reference standard of known starting concentration. As a consequence of this calculation, it is not possible to report the relative value of anti‐influenza IgA as µg; instead, it should be expressed as Unit/mL (U/mL), where 1 U corresponds to 1 µg of human IgA detected. It is important to run multiple samples collected from the same subject at different time points in the same ELISA plate.14 According to this method, the value of influenza‐specific IgA normalized through the total IgA content will be expressed as “(Influenza‐Specific IgA (U/mL)/Total IgA (µg/mL)) * 100.”.

### 4.2 Influenza HA‐Specific IgA and Total Protein

The basis of this method is the measurement of the total protein content in the samples (Figure 2). The determination of influenza HA‐specific IgA with respect to total protein generally proves to be the best choice when a large number of samples have to be evaluated, since it is easy to use, sensitive, and rapid.^75^ Depending on the total protein concentration obtained, two different methods of calculations can be adopted (Figure 2).

The first method will be applied if the total protein of the samples is higher than or equal to 1 mg/mL by standardization of nasal or saliva samples to a defined total protein content (Figure 2) which may vary according to the type of sample (nasal wash vs nasal swab vs saliva). The influenza‐specific IgA antibody titer is then calculated as the reciprocal of the highest dilution that yields an OD signal greater than or equal to a predefined cutoff value. However, since completely negative human nasal samples are usually not available, the exact calculation of a cutoff may not be optimal and require alternative approaches in the future.

One approach may be to use the “limit of blank” according to the following formula: “*Average of background signals (OD_Blank_) plus 2 standard deviations.”* 72 In this case, the cutoff value will be calculated without the need for a specific human sample; only ELISA reagents will be added to the coated plate together with the influenza antigen, and the background signal will be used to calculate the cutoff. An alternative possibility is the calculation of the cutoff value as the reciprocal of the highest dilution that shows an absorbance value >0.2 of the OD value after subtraction of the background as previously described.^73^

In the case of low total protein concentrations of the samples in general or of big differences of the total protein content of different test samples, an alternative approach, combining the two approaches described above, can be used which is based on the estimation of IgA content by using the ratio between the titer and the total IgA content (Figure 2).

## 5 NEUTRALIZING (NT) ANTIBODIES IN NASAL WASH/SWAB AND FUTURE ASSAYS

Some recent studies^74^, ^76^ have assessed neutralization (NT) antibody levels in standardized nasal wash/swab samples after intranasal immunization since NT antibodies are generally considered more specific than hemagglutination inhibition (HI) antibody titers in children vaccinated with LAIVs. However, it has been shown in a previous pediatric study with LAIV that influenza virus–specific salivary IgA levels correlated with serum HI responses,^76^ although it is also discussed that the measured HI titers may underestimate the protective potential of LAIVs.^60^, ^77^ NT antibodies in serum samples are usually assessed by means of the microneutralization (MN), either CPE (cytopathic effect)‐based^74^ or ELISA‐based,^37^ or the plaque‐reduction neutralization (PRNT) assay. In the present review, we focused on the CPE‐based MN assay, since this is the preferred method because of its simplicity of execution, its ability to evaluate large numbers of samples, and the standardization of the quantity of virus used in the assay.^78^ Along with the ELISA sIgA assay, the MN assay constitutes a valid approach to evaluate the immunogenicity of LAIVs, IIVs, or recombinant influenza vaccines (eg rHA) in inducing selective anti‐influenza antibodies with influenza virus–neutralizing potential.

Beside classical ELISA‐based and NT assays specific anti‐HA influenza antibodies, there are newer assays with increased precision and sensitivity, such as the XMAP (x = analyte MAP = Multi‐analite profiling) technology adapted for Luminex‐based IgA assays.^79^, ^80^ The XMAP technology is a serological method that can be applied to measure multiple proteins or antibodies in a single‐well reaction with high accuracy and reproducibility. The Luminex XMAP technology is based on the combination of different well‐established techniques such as flow cytometry, carboxylated microspheres, laser, and traditional chemistry. Briefly, specific nano‐magnetic beads can be coated with different purified proteins arising from the same pathogen or from different ones and then incubated with serum samples. Using specific biotinylated secondary antibody, the presence of antigen‐specific antibodies in sera can be easily measured and quantified by a dedicated detection system (Luminex). Wang et al^81^ applied this novel method for the simultaneous detection of antibodies against the Newcastle disease and avian influenza virus and have shown that the Luminex XMAP‐based assay has been up to 1024 times more sensitive for avian influenza virus antibody detection compared to the conventional ELISA assay. The minimal volume of sample required, the cost reduction for multiple detection in comparison with the classical methods, and the possibility to perform a rapid multiplexing in a single reaction are additional advantages of this new technology. However, these new generations of serological assays are not standardized and require further studies for the generation of validated and reproducible results. Currently, only the ELISA is a reliable and valuable approach to determine sIgA in various biological samples.

## 6 CONCLUSIONS

Influenza vaccines elicit protective immunity before a new influenza virus variant is able to spread; they therefore constitute a primary protection tool. Although the main protective effectors against influenza virus infection are CTLs, IgG, and IgA located in the respiratory mucosa, most of the vaccines currently available are inactivated vaccines that are administered via parenteral injection, and which mostly promote serum IgG rather than mucosal IgA (rev. in^82^, ^83^, ^84^). The importance of intranasally applied LAIVs is their ability to reproduce a natural infection without causing disease or virus transmission. They mimic the natural encounter with the antigen by activating the innate immune system and promoting antibody and T cell–mediated immune responses. This type of vaccine can induce a broader immune response in children than intramuscular vaccines.^31^, ^85^, ^86^ Furthermore, mucosal vaccines can elicit cross‐reactive antibodies in humans. However, the development of cross‐protective T lymphocytes has been observed in animal models, but this has not yet confirmed in humans.^85^, ^86^

An additional advantage of LAIVs is their consumer‐friendly needle‐free intranasal application which represents a minimal invasive delivery method, and it is expected with higher production capacities and a more widely distribution. For these reasons, its expanded use could increase the influenza vaccination coverage globally. Furthermore, it may represent a favorable approach for mass immunizations, especially in younger children since its application is not associated with pain.^87^

Although LAIVs have been on the global market for many years, no established correlates of protection for them are yet available.^77^ Moreover, previously reported discrepancies of efficacy data from Europe and the US further complicate the understanding of the immune response elicited by LAIV.^77^ Despite these complications, great efforts have been made in the recent years to develop novel intranasally administered vaccines to promote influenza virus‐specific sIgA,^30^, ^88^ which, as has been widely reported, provide broader protection than serum IgG. A robust mucosal response is fundamental in order to protect both the single individual and the entire population by preventing transmission of the virus to susceptible subjects.^89^ Notably, the use of the ELISA assay for IgA detection could play a major role in the evaluation of vaccine efficacy or effectiveness in the field, as currently influenza vaccine efficacy is traditionally assessed by means of serological assays that detect influenza‐specific serum antibodies induced by the vaccine itself. However, these assays cannot be properly applied to intranasal vaccines, which mainly induce local immune responses (rev. in^90^).

In conclusion, the measurement of sIgA in mucosal secretions for the evaluation of influenza vaccine efficacy or effectiveness and, in addition, also of the effectiveness of vaccines against other respiratory virus infections of the respiratory mucosae, is arousing great interest and may constitute a valuable asset.

## CONFLICT OF INTEREST

There are no conflicts of interest in the conduction of this study.

## AUTHOR CONTRIBUTIONS

EG and A.M involved in writing, reviewing, and editing processes and prepared the images; O. K., CT, and I.M involved in reviewing and editing processes; and EM involved in supervision and review process.
